# A New, Portable Orofacial Manometer for Measuring Tongue Strength and Endurance in Children: Laboratory-Based Validity Study

**DOI:** 10.2196/68967

**Published:** 2025-05-26

**Authors:** Rizky Kusuma Wardhani, Luh Karunia Wahyuni, Widjajalaksmi Kusumaningsih, Sarworini Bagio Budiardjo, Prasandhya Astagiri Yusuf, Sri Mardjiati Mei Wulan, Aria Kekalih, Titis Prawitasari, Sawitri Darmiati, Boya Nugraha

**Affiliations:** 1 Doctoral Program in Medical Sciences Faculty of Medicine Universitas Indonesia, Jl. Salemba Raya, Jakarta, 10430, Indonesia, 62 8195174942; 2 Department of Physical Medicine and Rehabilitation, Faculty of Medicine Universitas Indonesia-Dr. Cipto Mangunkusumo Hospital, Jakarta, Indonesia; 3 Department of Pediatric Dentistry, Faculty of Dentistry, Universitas Indonesia, Jakarta, Indonesia; 4 Department of Medical Physiology and Biophysics / Medical Technology IMERI, Faculty of Medicine Universitas Indonesia, Jakarta, Indonesia; 5 Department of Physical Medicine and Rehabilitation, Dr. Soetomo General Academic Hospital, Surabaya, Indonesia; 6 Department of Physical Medicine and Rehabilitation, Faculty of Medicine Universitas Airlangga, Surabaya, Indonesia; 7 Community Medicine Department, Faculty of Medicine Universitas Indonesia, Jakarta, Indonesia; 8 Department of Pediatric, Faculty of Medicine Universitas Indonesia-Dr. Cipto Mangunkusumo Hospital, Jakarta, Indonesia; 9 Department of Radiology, Faculty of Medicine Universitas Indonesia-Dr. Cipto Mangunkusumo Hospital, Jakarta, Indonesia; 10 Department of Rehabilitation Medicine, Hannover Medical School, Hannover, Germany

**Keywords:** pediatric swallowing, tongue strength, tongue endurance, orofacial manometer, validity

## Abstract

**Background:**

An accurate tongue strength and endurance assessment is necessary for pediatric dysphagia. TongueFit is a new, portable orofacial manometer for measuring tongue strength and endurance and a game-based training app for children.

**Objective:**

This study aimed to test the validity of TongueFit compared with a standard manometer as the current gold standard for measuring air pressure.

**Methods:**

This laboratory study compared TongueFit and a standard manometer as the gold standard for measuring air pressure. This study was conducted in 3 different experimental conditions. The first experiment compared TongueFit and the standard manometer using a force tester (MCT-2150), with pressure controlled by MSatLite software. The second and third experiments involved 2-cm and 3-cm bulbs between the 2 devices. This study used Lin concordance correlation to measure the level of agreement.

**Results:**

There was a mean absolute difference of 0.005 kilopascals (kPa) between the TongueFit and the standard manometer (n=35, ρC=1). Statistical analysis showed perfect agreement correlation (ρC=1). By using the 2-cm bulb, TongueFit’s mean was 0.007 kPa lower, also showing perfect agreement (ρC=1). Furthermore, using the 3-cm bulb, results showed almost perfect agreement (ρC=0.999) with the TongueFit’s mean, which was 0.044 kPa lower.

**Conclusions:**

This study confirms the high validity of TongueFit as an orofacial manometer compared with a standard manometer, with negligible mean differences, as well as near-perfect and perfect agreement in the experiments. These results confirm that TongueFit is a valid and accurate tool for assessing tongue strength.

## Introduction

Eating and swallowing are dynamic processes essential for children’s growth and development [1]. This complex mechanism involves the coordination of more than 30 nerves and muscles in the oral cavity, pharynx, larynx, and esophagus [2,3]. Typically, the swallowing process is divided into 3 distinct phases based on the passage of the bolus through different anatomical regions: the oral phase, the pharyngeal phase, and the esophageal phase. Each phase is critical in facilitating food and fluids’ safe and efficient passage from the mouth to the stomach, ensuring proper nutrition and hydration for optimal growth and function [3-5].

The tongue comprises bundles of highly flexible, vascularized, and innervated muscles that play a critical role in the oral phase of swallowing. Tongue movements, controlled by the intrinsic and extrinsic muscles, include protrusion, retraction, elevation, depression, rotation, lateralization, cupping, and twisting [6,7]. Tongue dysfunction can result in reduced tongue strength and endurance, potentially compromising swallowing function and leading to difficulty eating and swallowing, a condition commonly referred to as oral dysphagia [8]. Reduced tongue strength can significantly impact several aspects of tongue function, including chewing, bolus control, and bolus clearance. Previous research suggests that reduced tongue strength may contribute to oral and pharyngeal dysphagia [9]. Tongue muscle weakness could decrease intraoral pressure during swallowing, thereby increasing pharyngeal residue and risk of aspiration in the pharyngeal phase [6,10].

The early diagnosis of dysphagia is important for the prevention of malnutrition, dehydration, and aspiration pneumonia, in addition to allowing adequate treatment [11]. Previous studies evaluating neurological pathologies that affect swallowing disorders have observed a positive association between tongue strength and dysphagia. Dysphagic patients had significantly reduced tongue strength and endurance compared with patients with preserved swallowing [11-13]. Therefore, evaluation of tongue strength and endurance is critical, especially in populations of children who have feeding difficulties and are at increased risk for eating and swallowing disorders, such as children with cerebral palsy, Down syndrome, and other related conditions [14,15].

Assessment of tongue strength is essential because of its important role in eating and swallowing [16]. Various orofacial manometers have been developed to measure tongue force objectively and continue to evolve. One of the most widely used orofacial manometers is the Iowa Oral Performance Instrument (IOPI). The IOPI is a portable device designed to measure tongue strength and endurance, using an air-filled bulb as a sensor to measure the maximum isometric force of the tongue in kilopascals (kPa). The IOPI provides biofeedback in the form of a vertical light indicator that illuminates when the target pressure is reached. It also includes a training mode designed to improve tongue strength and endurance [17-19]. It has been used extensively in numerous studies and has shown reasonable validity and reliability. However, the IOPI has limitations. It is unavailable in the Indonesian market, and users reported problems slipping the air-filled bulb during measurements.

There was no tool specifically designed for children to measure and train their tongue strength and endurance. Therefore, we developed a device called “TongueFit” that is designed for both clinical and home use. It features a nonslip surface on the air-filled bulb sensor and incorporates a game-based biofeedback for exercise. The biofeedback was created as an Android-based app to complement the device. This app allows users to access measurement results, historical data, and game-based exercises. Integrating game-based biofeedback in TongueFit enhances dysphagia treatment by providing real-time feedback and rewarding good performance with scores. This approach transforms therapy into an engaging experience, fostering greater participation, particularly in pediatric patients.

Although TongueFit can also be used as an exercise tool to improve tongue strength and endurance, the focus of this study was solely on assessing the validity of TongueFit as a measurement tool. By focusing solely on the validity of TongueFit’s measurement capabilities, we seek to provide empirical evidence to support its efficacy as a tool for assessing tongue strength and endurance. This study aimed to determine the accuracy of TongueFit in quantifying tongue muscle performance in kPa. In addition, by confirming the validity of TongueFit’s measurements, we aimed to establish TongueFit as a valuable tool in clinical settings, facilitating more accurate diagnoses and tailored treatment plans for individuals with tongue-related impairments.

## Methods

### Overview of TongueFit

**Figure 1.**
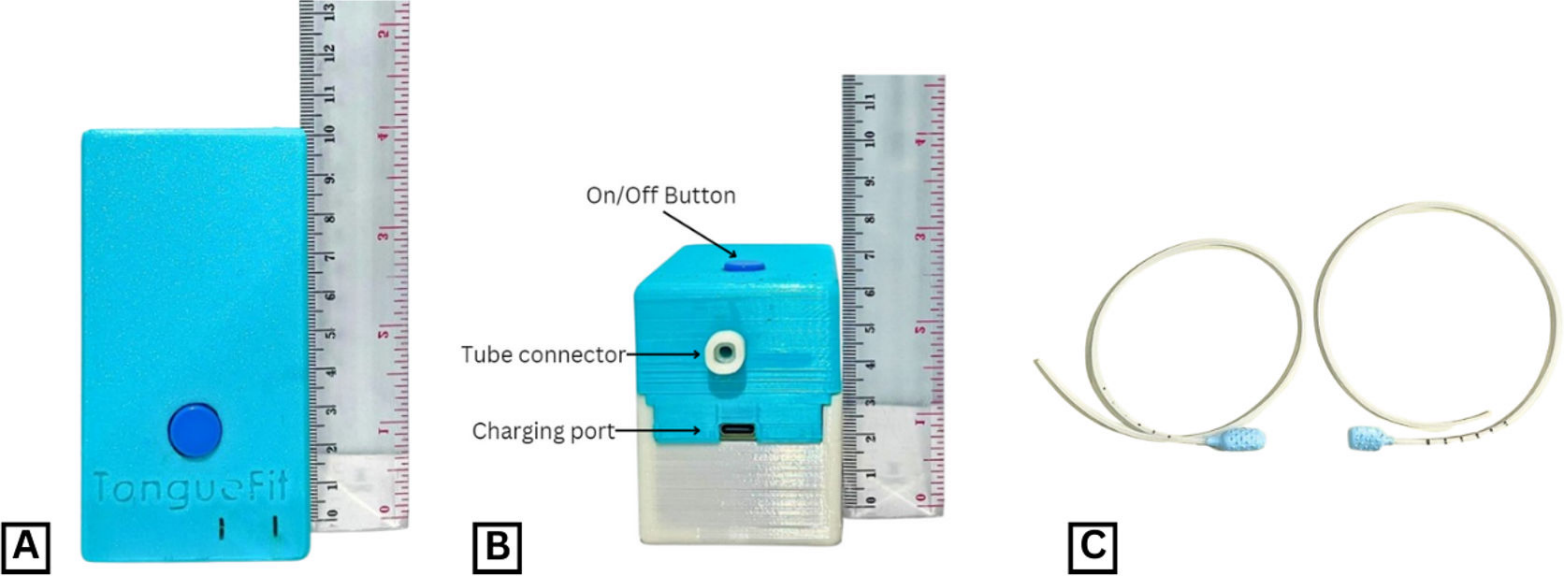
The hardware components of TongueFit. (A) The manometer; (B) the body of the TongueFit comprises the manometer hardware, an on/off button, a tube connector, and a charging port; and (C) the tongue bulb has two different sizes, consisting of the 2-cm bulb (right) and the 3-cm bulb (left).

TongueFit consisted of 2 essential components: hardware and software, both of which were interdependent for operation. The hardware included a manometer device along with 2 variations of bulb sizes. The bulbs and tubes were made of biobased polyurethane and polylactic acid—food-grade materials that had undergone rigorous toxicity testing in the laboratory to ensure their safety (Figure 1). The tests were performed at the Stem Cells and Tissue Engineering Research Centre, Cluster Indonesian Medical Education and Research Institute Fakultas Kedokteran Universitas Indonesia, using human stem cells. A material was declared toxic if the percentage of stem cell death exceeded 30% of the total stem cells within the control group in the tests. Based on the toxicity tests conducted on the bulb and hose materials, which were repeated at 15, 30, and 60 minutes, the percentage of cell death was less than 16%. Therefore, it can be concluded that the bulbs and tubes are nontoxic.

**Figure 2.**
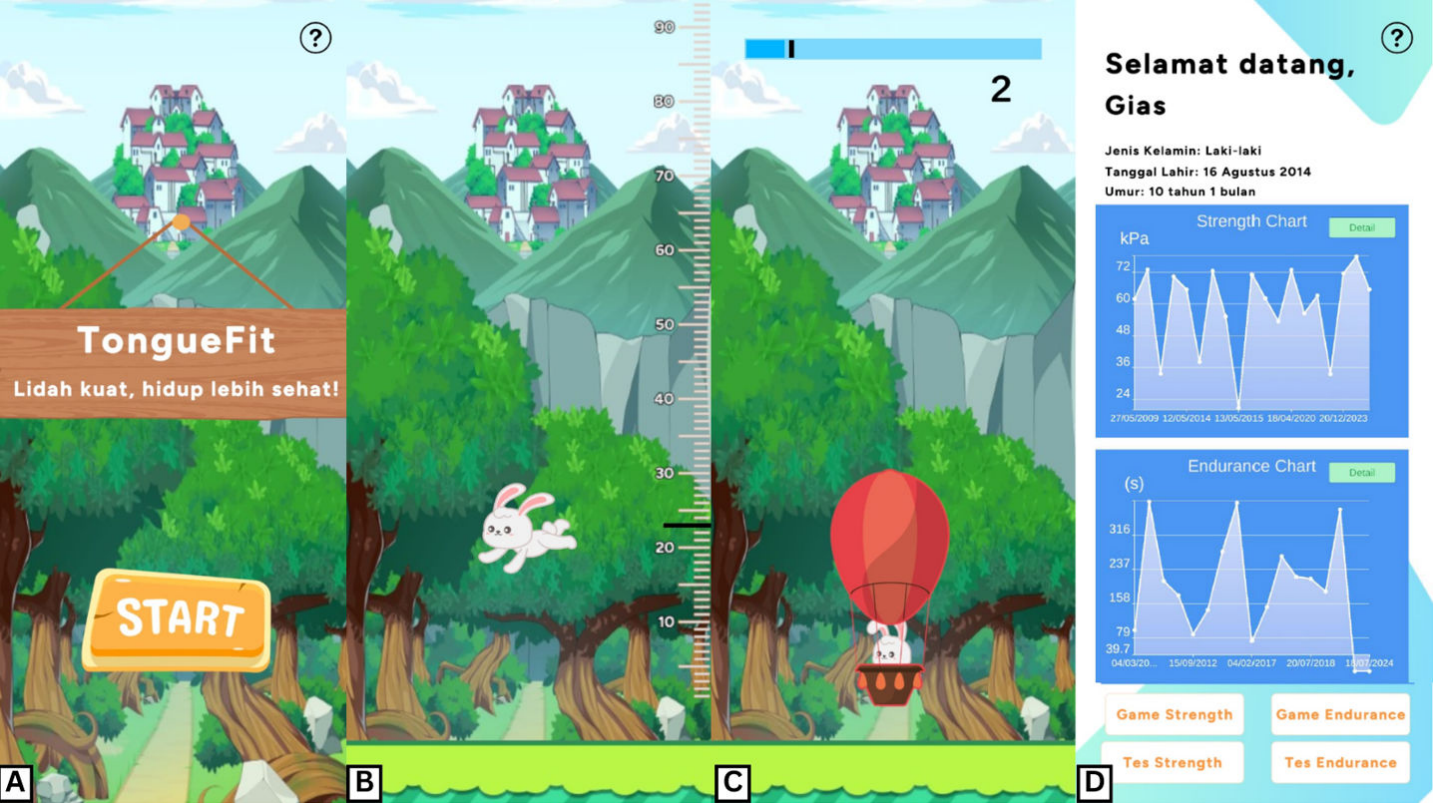
The TongueFit app. (A) Opening page; (B) tongue strength test; (C) tongue endurance test; and (D) tongue strength and endurance chart result.

The software component included a mobile app that allows users to measure tongue strength and endurance, access measurement results, review historical data, and participate in game-based exercises in Bahasa (Figure 2). Tongue strength is assessed by positioning the bulb anteromedial on the tongue and instructing the patient to elevate the tongue against the palate with maximum effort. This measurement is performed 3 times, and the highest recorded value is used as the reference target for endurance assessment and exercise therapy. Tongue endurance is evaluated by having the patient sustain tongue elevation against the palate at 40%‐60% of their maximum strength for as long as possible.

Tongue strength and endurance measurements were conducted before therapy to establish baseline values, which served as a reference for the exercise prescription using “TongueFit.” The strengthening and endurance exercises targeted the anterior tongue, considering its muscle fiber composition, primarily type IIa fibers, which exhibit fast contraction properties while maintaining a degree of endurance [9,20]. As a result, the exercise protocol incorporated both strengthening and endurance components. The exercise prescription adhered to the FITT (frequency, intensity, time, and type) principle, with strengthening exercises performed at an intensity of 60%‐80% and endurance exercises at 40%‐60% of maximum tongue strength. Each session lasted for 15 minutes per exercise type, with 30 repetitions per session.

The development of TongueFit’s device and app underwent user experience testing through the creation of a mock-up [21]. The mock-up of the tool was tested on a group of medical doctors experienced in pediatric rehabilitation representing potential users to ensure the quality and comfort of the final product. The testing process for the mock-up of TongueFit’s device focused on evaluating comfort and ergonomics and identifying potential design improvements. In parallel, TongueFit’s app mock-up trial assessed navigation, design consistency, and user interface comprehension. Based on the test results, the mock-up was improved and adjusted to better meet the needs of the target users.

**Figure 3.**
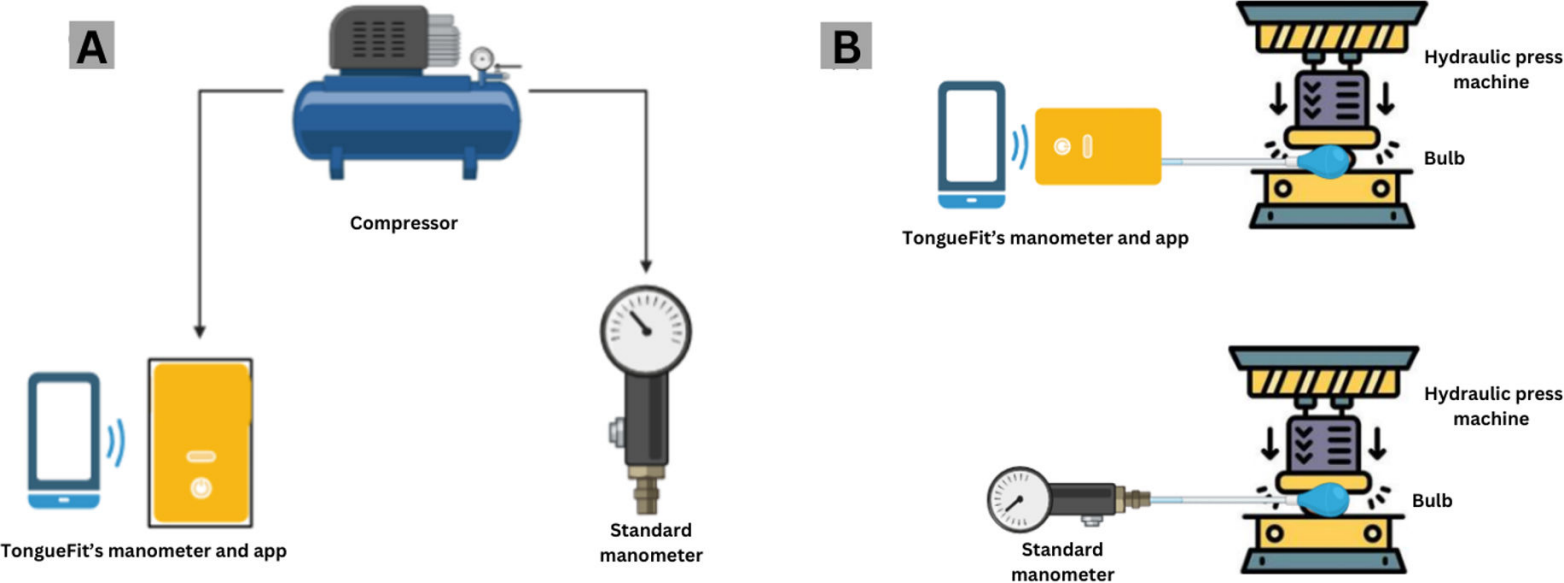
Validation test scheme. (A) TongueFit versus standard manometer with compressor, and (B) TongueFit versus standard manometer with bulb validation test.

In this study, we compared measurements obtained from TongueFit and a standard manometer (SNDWAY SW-512C, Dongguan Sndway Electronic), considered the gold standard for measuring air pressure differences. TongueFit offered two different bulb sizes: a 2-cm bulb for children under 6 years of age and a 3-cm bulb for children over 6 years of age and adults. Our experiment included the evaluation of both bulb sizes and the TongueFit device itself. This study was conducted in 3 different experimental conditions (Figure 3). The first experiment compared TongueFit and the standard manometer using a compressor. The second and third experiments involved 2-cm and 3-cm bulbs between the 2 devices.

### TongueFit Versus Standard Manometer

This experiment was designed to validate the ability of the TongueFit to provide accurate pressure measurements compared with the standard manometer. Each device was connected to a universal high-performance force tester (MCT-2150, A&D), which acted as an air compressor and controlled air pressure source. This connection was made alternately through a polyurethane tube. The air pressure given to each device was controlled by MSatLite software (A&D). The pressure was gradually increased, starting at 2 kPa and increasing by 2 kPa for each measurement to a maximum of 70 kPa. Each measurement started from 0 kPa.

### TongueFit With 2-cm Bulb Versus Standard Manometer With 2-cm Bulb

This experiment aimed to ensure the consistency of measurement results between the TongueFit and the standard manometer when each device was connected to a 2-cm air-filled bulb that we developed. To maintain a controlled pressure on the bulb, the previously used force-tester device (MCT-2150, A&D) was configured as a computer-controlled hydraulic press machine in the laboratory, using software called MSatLite (A&D) to apply pressure on the bulb.

To conduct this experiment, TongueFit was connected to the air-filled bulb via a tube, and the bulb was positioned under the hydraulic press machine with a fixture. The machine applied pressure to the entire surface of the bulb to ensure uniform pressure distribution. The compression of the bulb was carried out by using a hydraulic machine, starting from 0.6 mm and increasing by 0.2 mm for each measurement until 5 mm was reached, each measurement starting from 0 kPa. The same procedure was then performed with the 2-cm bulb connected to the standard manometer.

### TongueFit With 3-cm Bulb Versus Standard Manometer With 3-cm Bulb

This experiment followed identical procedures to the 2-cm bulb experiment. Maintaining consistency in the experimental protocol ensured that the methodology, conditions, and variables studied remained consistent throughout the investigation. This approach allowed for a direct comparison between the results of the 2 experiments, facilitating a comprehensive evaluation of the effect of different bulb sizes.

### Statistical Analysis

The obtained measurement data were recorded and processed using Microsoft Excel. Data analysis was performed using SPSS software (version 27; IBM Corp). The validity test used the Lin concordance correlation coefficient to assess the degree of agreement between the standard manometer and TongueFit. Lin concordance correlation coefficient (ρC) results of <0.9 indicate poor validity, the range of 0.9‐0.949 indicates moderate validity, 0.95‐0.99 indicates substantial validity, and values >0.99 indicate almost perfect validity.

## Results

### Overview

This study was a laboratory-based study that compared TongueFit and a standard manometer in 3 different experiments.

### TongueFit Versus Standard Manometer

**Figure 4.**
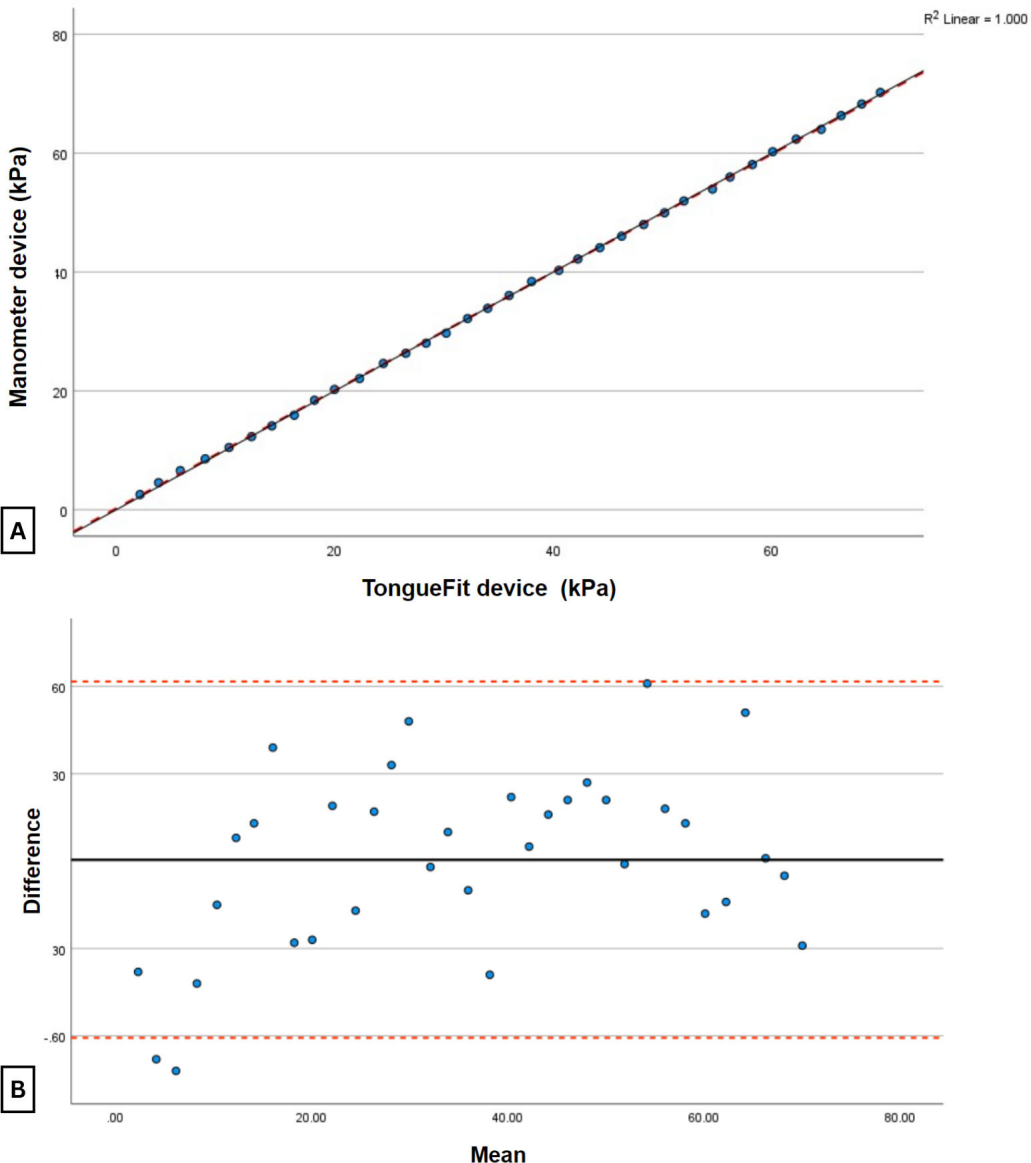
Relationship between TongueFit device readings and standard manometer device readings. (A) The graph of the TongueFit device is a scatter plot with a black solid line representing the best line and a red dashed line representing the perfect line, and (B) the Bland-Altman plot of the difference between the 2 devices, with the black solid line representing the mean difference between the measurements and the red dotted line representing 1.96 SDs above and below the mean difference.

The sensor readings obtained by TongueFit (Table 1 and Figure 4) show a slightly higher mean value of 0.005 kPa compared with those recorded by the standard manometer when the compressor administered air pressure. Upon further statistical analysis, a perfect agreement value (ρC=1) indicates a precise agreement between TongueFit and the standard manometer readings.

**Table 1.** The degree of conformity between TongueFit, the standard manometers, and the compressor.

Compressor (kPa)	TongueFit (kPa)	Standard manometer (kPa)
2	2.18	2.56
4	3.88	4.56
6	5.87	6.59
8	8.15	8.57
10	10.33	10.48
12	12.39	12.31
14	14.25	14.12
16	16.30	15.91
18	18.15	18.43
20	19.98	20.25
22	22.27	22.08
24	24.45	24.62
26	26.51	26.34
28	28.36	28.03
30	30.20	29.72
32	32.16	32.18
34	34.00	33.9
36	35.96	36.06
38	38.02	38.41
40	40.51	40.29
42	42.25	42.2
44	44.26	44.1
46	46.26	46.05
48	48.27	48
50	50.18	49.97
52	51.95	51.96
54	54.57	53.96
56	56.17	55.99
58	58.22	58.09
60	60.08	60.26
62	62.23	62.37
64	64.52	64.01
66	66.34	66.33
68	68.23	68.28
70	69.93	70.22

### TongueFit With 2-cm Bulb Versus Standard Manometer With 2-cm Bulb

**Figure 5.**
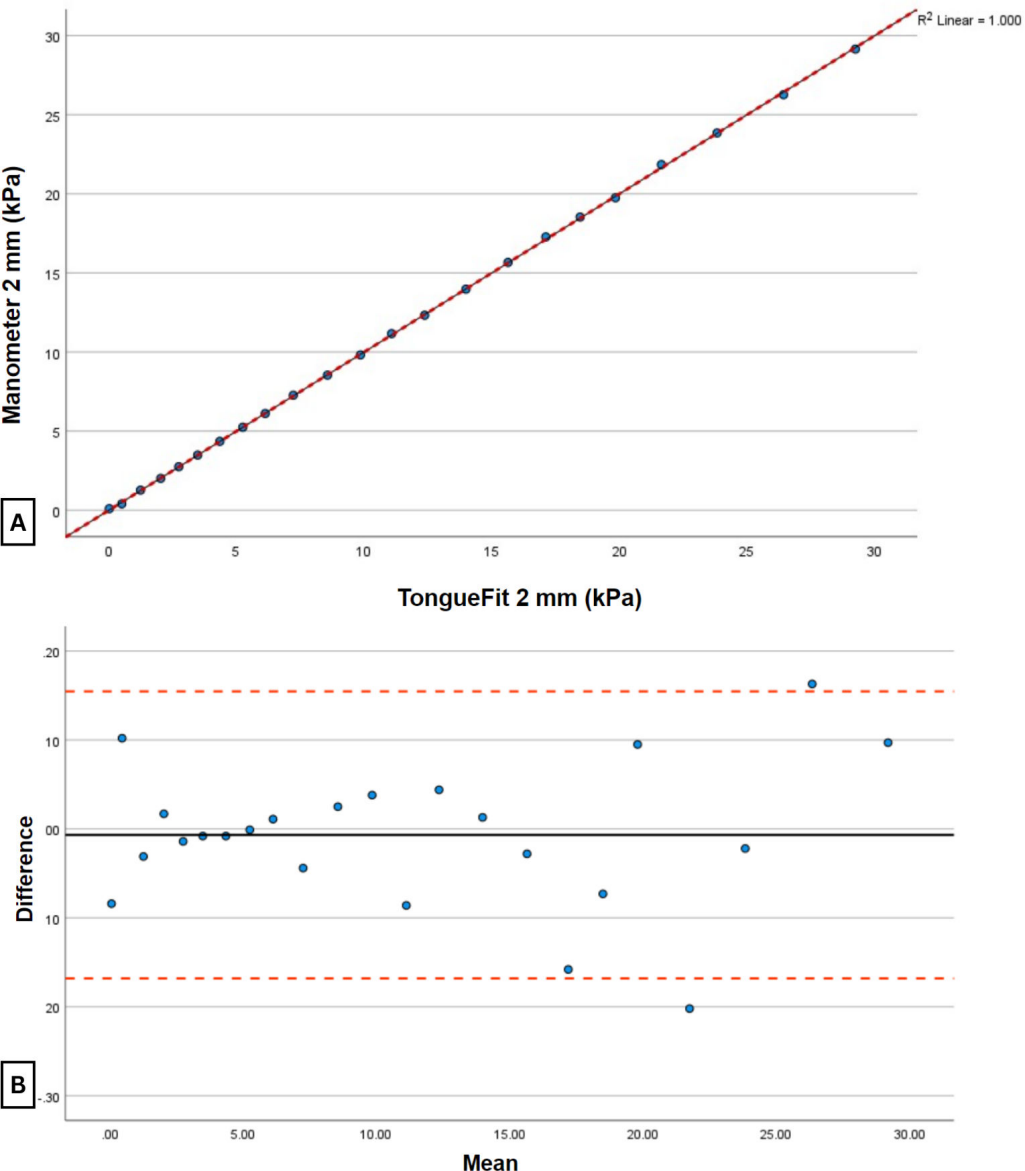
Relationship between TongueFit and standard manometer pressure readings connected to the same 2-cm bulb. (A) The scatter plot with the black solid line representing the line of best fit and the red dashed line representing the line of perfect agreement. (B) The Bland-Altman plot of the differences between the 2 devices, with the black solid line representing the average differences between the measurements and the red dashed line representing 1.96 SDs above and below the average differences.

When tested with the 2-cm bulb (Table 2 and Figure 5), TongueFit showed a perfect agreement value (ρC=1), with its mean value being 0.007 kPa lower than that of the standard manometer. This indicated a consistent trend of TongueFit readings being slightly lower than the standard manometer across different bulb sizes.

**Table 2.** The degree of conformity between TongueFit and standard manometers with the 2-cm bulb.

Hydraulic (mm)	TongueFit (kPa)	Standard manometer (kPa)
0.6	0.02	0.1
0.8	0.51	0.41
1.0	1.24	1.27
1.2	2.03	2.02
1.4	2.74	2.76
1.6	3.48	3.49
1.8	4.35	4.36
2.0	5.25	5.25
2.2	6.13	6.12
2.4	7.23	7.27
2.6	8.57	8.54
2.8	9.86	9.82
3.0	11.08	11.16
3.2	12.37	12.33
3.4	13.99	13.98
3.6	15.64	15.67
3.8	17.12	17.28
4.0	18.46	18.54
4.2	19.85	19.75
4.4	21.65	21.85
4.6	23.83	23.85
4.8	26.43	26.27
5.0	29.25	29.15

### TongueFit With 3-cm Bulb Versus Standard Manometer With 3-cm Bulb

**Figure 6.**
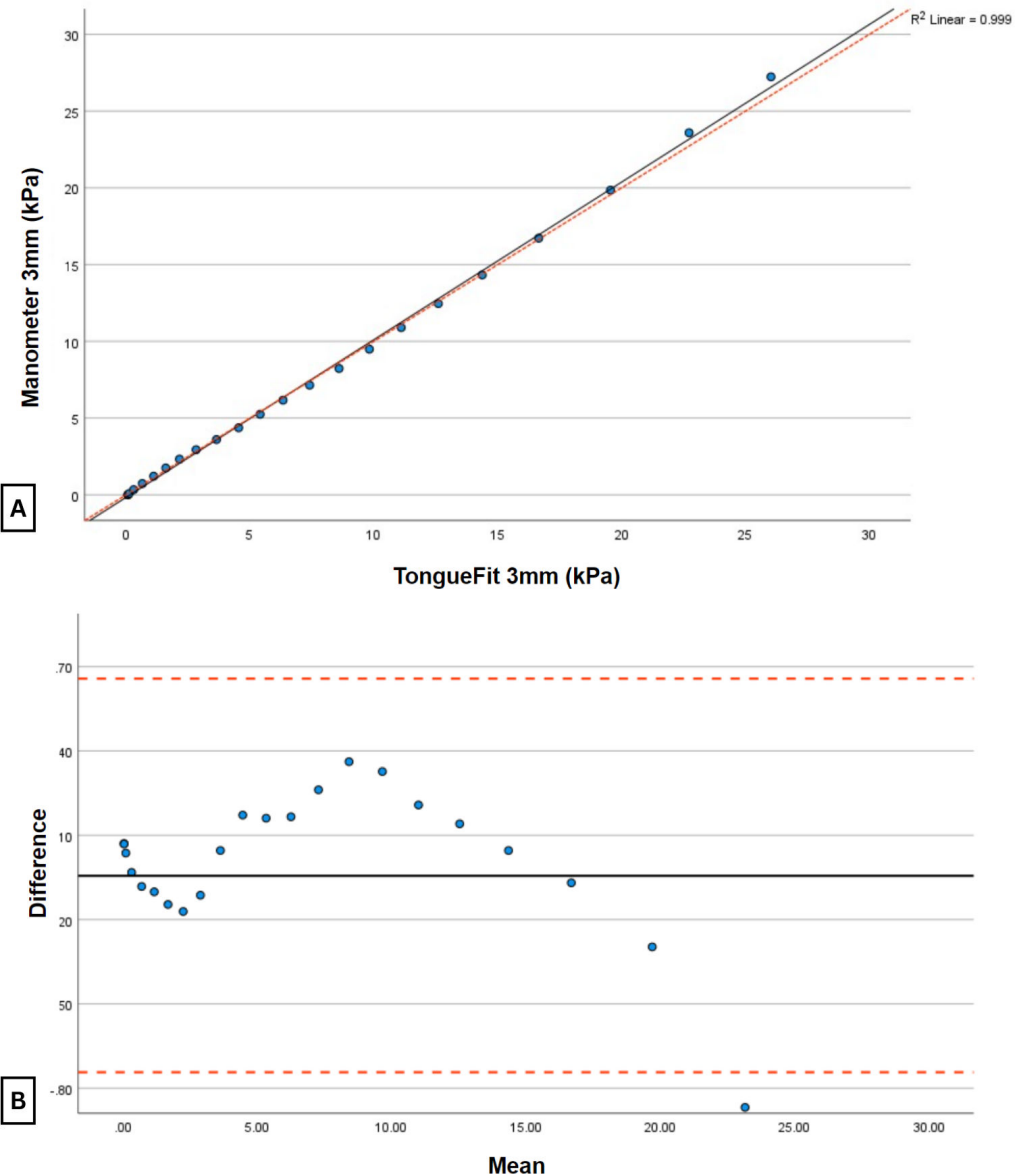
Relationship between TongueFit and standard manometer pressure readings connected to the same 3-cm bulb. (A) The scatter plot with the black solid line representing the line of best fit and the red dashed line representing the line of perfect agreement. (B) The Bland-Altman plot of the differences between the two devices, with the black solid line representing the average differences between the measurements and the red dashed line representing 1.96 SDs above and below the average differences.

The test results using the 3-cm bulb (Table 3 and Figure 6) showed almost perfect agreement (ρC=0.999), with a 95% CI ranging from 0.998 to 0.999. In this scenario, the average TongueFit reading was significantly lower than the standard manometer by 0.044 kPa.

Overall, TongueFit showed strong validity across all experiments, as indicated by ρC values greater than 0.99. This means an almost perfect agreement between the TongueFit measurements and those obtained with the standard manometer.

**Table 3.** The degree of conformity between TongueFit and standard manometers with the 3-cm bulb.

Hydraulic (mm)	TongueFit (kPa)	Standard manometer (kPa)
0.6	0.07	0.00
0.8	0.08	0.01
1.0	0.12	0.09
1.2	0.31	0.34
1.4	0.66	0.74
1.6	1.11	1.22
1.8	1.6	1.75
2.0	2.16	2.33
2.2	2.83	2.94
2.4	3.65	3.6
2.6	4.55	4.37
2.8	5.41	5.25
3.0	6.34	6.17
3.2	7.41	7.15
3.4	8.6	8.24
3.6	9.82	9.49
3.8	11.11	10.9
4.0	12.61	12.46
4.2	14.38	14.33
4.4	16.66	16.73
4.6	19.56	19.85
4.8	22.73	23.59
5.0	26.03	27.24

## Discussion

### Principal Findings

Assessment of tongue strength is essential because of its important role in eating and swallowing [13]. Over time, several methods have been developed to measure tongue strength to evaluate tongue function. The orofacial manometer is a widely used tool for quantifying tongue strength, with the IOPI being one of the most commonly used devices. When lingual force is applied to the air-filled bulb, this measures the pressure difference against a predetermined air volume. It then digitally displays the pressure inside the bulb in kPa [10,11]. The TongueFit device, which we developed, works on the same principle. In addition, we integrated game-based biofeedback into the exercises to increase the children’s engagement and participation in the process. This study provided evidence of the satisfactory validity of TongueFit compared with the standard manometer. In the first experiment, the comparison between TongueFit and the standard manometer showed a negligible mean difference of 0.005 kPa, indicating a high level of agreement between the measurements obtained by both devices. In addition, the analysis showed a perfect agreement value (ρC=1), further emphasizing the accuracy of the TongueFit measurements. The analysis of the Bland-Altman plot also suggested a consistent, perfect agreement using the 2-cm bulb (ρC=1) and the 3-cm bulb (ρC=0.999). This study confirms the high validity of TongueFit as an orofacial manometer compared to the standard manometer, with near-perfect and perfect agreement in 3 experiments.

Previously developed tongue measurement devices, such as the Tongueometer and the Tongue Pressure Meter (TPM-01; JMS), had used the IOPI as a gold standard to validate or compare their measurement results [22,23]. Unlike previous studies, we refrained from this approach primarily due to its unavailability in Indonesia and the lack of published validation studies for the IOPI. Instead, we used the standard manometer, a standard device used for measuring pressure differentials, similar to the IOPI’s air-filled bulb principle. Consequently, we considered the standard manometer an accurate and valid alternative for assessing air pressure differentials, similar to the IOPI’s tongue force measurement method.

In contrast to this study, where the TongueFit was compared with a standard manometer, other studies have used the IOPI as a reference tool for comparison. Gibbons et al [24] compared the Tongueometer and the IOPI in measuring lingual pressure. Statistically significant, positive correlations were found between the mean values measured by the 2 devices for anterior isometric lingual pressure (concordance correlation coefficient=0.74, *P*<.001), demonstrating the concurrent validity of the Tongueometer with the IOPI. The independent samples *t* test revealed no statistically significant difference in the means for anterior isometric lingual pressure (*t*_74_=−0.5, *P*=.65), suggesting similar performance between the Tongueometer and IOPI in this regard. However, a study by Curtis et al [22] found that Tongueometer pressure readings under controlled conditions (without using a bulb) were typically 7 kPa lower on average than IOPI pressure readings. Lin concordance correlation was used to assess the validity of Tongueometer readings compared with the IOPI and showed substantial validity (ρC=0.97, 95% CI 0.961‐0.977). In addition, analysis using a Bland-Altman plot showed that only 16.8% of the data points were in agreement.

Yoshikawa et al [23] used the Pearson correlation coefficient to compare the measurement results of the TPM-01 and IOPI. Their results showed significant correlations between TPM-01 and IOPI measurements in both female participants (*R*^2^=0.74, *P*<.01; IOPI PRO=TPM-01×1.21+11.2) and male participants (*R*^2^=0.64, *P*<.01; IOPI PRO=TPM-01×1.07+15.4). Compared with the Tongueometer and the TPM-01, TongueFit showed better measurement validity than both devices. Thus, TongueFit can be considered a valid tool for assessing tongue strength, providing accurate measurements comparable with those obtained with the standard manometer under different experimental conditions. However, it was important to note that TongueFit was validated using the standard manometer, not the IOPI.

Most studies on tongue measurement devices focus on adult populations, while only a few target pediatric populations. Potter and Short [25] used the IOPI device to measure tongue strength in typically developing children aged 3‐16 years old using a standard IOPI bulb. One concern in Potter and Short’s study was whether younger children could tolerate the IOPI bulb since it contacts more than 50% of the tongue surface in a 3-year-old child compared with only 30% in adults. While they found that most younger children tolerated the bulb well, there were still a few who did not. Given the small sample size of the age group in the study (n=28), it was possible that with a larger sample size, more children would not be able to tolerate the light bulb. To the best of our knowledge, no device has been specifically designed for children. Previous studies used commonly available tools, whereas the TongueFit used in this study was specifically designed for children and incorporated a specialized app tailored to their needs. Therefore, this study is essential for advancing future clinical applications. Furthermore, assessing the validity of the orofacial manometer and the bulb is important.

Curtis et al [22] evaluated the tongue bulb of the Tongueometer against that of the IOPI, using both the IOPI and Tongueometer devices for separate measurements. In both sets of measurements, the Tongueometer consistently registered higher pressure readings. Specifically, when measured with the IOPI, the bulb of the Tongueometer had a median pressure of 1 kPa higher than that of the IOPI, increasing to 4 kPa higher when pressures exceeded 40 kPa. Analysis using Lin concordance correlation underscored substantial validity between the bulb of the tongueometer and the bulb of the IOPI (ρC=0.987, 95% CI 0.982‐0.991). Conversely, the pressure readings of his bulb exceeded those of the IOPI’s bulb by 2.7 kPa when measured with the tongueometer, with the discrepancy increasing with increasing pressure. Lin concordance correlation between the bulb of the tongueometer and the bulb of the IOPI (ρC=0.992, 95% CI 0.988‐0.9914) indicated substantial to excellent agreement. Although the exact cause of this variance remains unclear, it is speculated to be due to differences in the size and shape of the tongue bulbs. In this study, the TongueFit showed better results according to Lin concordance correlation, showing a perfect agreement value for the 2-cm bulb (ρC=1) and an almost perfect agreement value for the 3-cm bulb (ρC=0.999).

To our knowledge, no other studies have evaluated the validity of tongue bulb measurements. A previous study compared the performance of the Tongueometer bulbs and the IOPI bulbs [22]. In contrast, our investigation sought to elucidate the differences in pressure determinations between a standard manometer and the TongueFit when using the TongueFit bulb. This thorough investigation was conducted to verify the consistency of pressure readings between the TongueFit and the standard manometer, with both devices connected to the 2-cm and 3-cm air-filled bulbs developed in our study.

Since the TongueFit bulb would directly contact oral cavity structures, ensuring its safety is paramount. Therefore, we conducted a toxicity assessment using human stem cells to evaluate the raw materials comprising the bulb, tongue bulb, and tube. Our results indicate that the raw material, bulb, and tube have nontoxic properties, confirming their safety for intraoral use. This tool and app also passed preliminary testing and evaluation of the mock-up to identify potential improvements and enhancements to increase the functionality of both the device and the app. Therefore, the final tool and app are well-aligned with user needs, incorporating the findings from the mock-up evaluation. As we mentioned earlier, this study focused solely on the validity of TongueFit’s measurement capabilities. The effect of TongueFit as a tool for tongue strength and endurance exercises will be investigated in future studies.

### Conclusion

In conclusion, this study underlined the satisfactory validity of TongueFit compared with the standard manometer. The first experiment showed a negligible mean difference between TongueFit and the standard manometer, with a perfect agreement value, indicating high accuracy in TongueFit measurements. In addition, in the 2-cm and 3-cm bulb experiments, TongueFit showed a perfect agreement value compared with the standard manometer. This consistent trend suggests that TongueFit measurements are slightly lower across different bulb sizes. In addition, the test results using the 3-cm bulb showed an almost perfect agreement value. Furthermore, TongueFit has passed preliminary testing and toxicity assessments, ensuring it is safe, comfortable, and suitable for users. These results established TongueFit as an accurate and valid tool for assessing tongue strength and endurance, providing results that closely match those obtained with the standard manometer.

## Abbreviations

FITT: frequency, intensity, time, and type
IOPI: Iowa Oral Performance Instrument
kPa: kilopascals
TPM: Tongue Pressure Meter

## References

[1] Kaiser L, Park T (2020). Feeding and swallowing development in children graduate independent studies. Commun Sci Disord.

[2] Matsuo K, Palmer JB (2008). Anatomy and physiology of feeding and swallowing: normal and abnormal. Phys Med Rehabil Clin N Am.

[3] Massey BT (2006). Physiology of oral cavity, pharynx and upper esophageal sphincter. GI Motil Online.

[4] Lang IM (2024). Coordination of pharyngeal and esophageal phases of swallowing. J Neurogastroenterol Motil.

[5] Pitts T, Iceman KE (2023). Deglutition and the regulation of the swallow motor pattern. Physiology (Bethesda).

[6] Dotiwala AK, Samra NS (2024). StatPearls [Internet].

[7] Kayalioglu M, Shcherbatyy V, Seifi A, Liu ZJ (2007). Roles of intrinsic and extrinsic tongue muscles in feeding: electromyographic study in pigs. Arch Oral Biol.

[8] van den Engel-Hoek L, de Groot IJM, de Swart BJM, Erasmus CE (2015). Feeding and swallowing disorders in pediatric neuromuscular diseases: an overview. J Neuromuscul Dis.

[9] Lin CJ, Lee YS, Hsu CF (2022). Effects of tongue strengthening exercises on tongue muscle strength: a systematic review and meta-analysis of randomized controlled trials. Sci Rep.

[10] Palmer JB, Drennan JC, Baba M (2000). Evaluation and treatment of swallowing impairments. Am Fam Physician.

[11] Hiraoka A, Yoshikawa M, Nakamori M (2017). Maximum tongue pressure is associated with swallowing dysfunction in ALS patients. Dysphagia.

[12] Lee JH, Kim HS, Yun DH (2016). The relationship between tongue pressure and oral dysphagia in stroke patients. Ann Rehabil Med.

[13] Borges AL de F, Velasco LC, Ramos HVL (2022). Association between dysphagia and tongue strength in patients with amyotrophic lateral sclerosis. Braz J Otorhinolaryngol.

[14] Serel Arslan S (2022). Swallowing related problems of toddlers with Down syndrome. J Dev Phys Disabil.

[15] Mouilly M, El Midaoui A, El Hessni A (2022). The effects of swallowing disorders and oral malformations on nutritional status in children with cerebral palsy. Nutrients.

[16] Park JS, Kim HJ, Oh DH (2015). Effect of tongue strength training using the Iowa Oral Performance Instrument in stroke patients with dysphagia. J Phys Ther Sci.

[17] Adams V, Mathisen B, Baines S, Lazarus C, Callister R (2013). A systematic review and meta-analysis of measurements of tongue and hand strength and endurance using the Iowa Oral Performance Instrument (IOPI). Dysphagia.

[18] Franciotti R, Di Maria E, D’Attilio M, Aprile G, Cosentino FG, Perrotti V (2022). Quantitative measurement of swallowing performance using Iowa Oral Performance Instrument: a systematic review and meta-analysis. Biomedicines.

[19] Pitts LL, Cox A, Morales S, Tiffany H (2022). A systematic review and meta-analysis of Iowa Oral Performance Instrument measures in persons with Parkinson’s disease compared to healthy adults. Dysphagia.

[20] Wilson JM, Loenneke JP, Jo E, Wilson GJ, Zourdos MC, Kim JS (2012). The effects of endurance, strength, and power training on muscle fiber type shifting. J Strength Cond Res.

[21] Baharum A, Amirul SM, Yusop NMM, Halamy S, Fabeil NF, Ramli RZ (2017). Advances in Visual Informatics.

[22] Curtis JA, Mocchetti V, Rameau A (2023). Concurrent validity of the IOPI and Tongueometer orofacial strength measurement devices. Laryngoscope.

[23] Yoshikawa M, Fukuoka T, Mori T (2021). Comparison of the Iowa Oral Performance Instrument and JMS tongue pressure measurement device. J Dent Sci.

[24] Gibbons T, Abrams SW, Mohsin N, Guastella R, Oppedisano S, Namasivayam-MacDonald A (2023). A pilot assessment of concurrent validity and comparative reference values for the Tongueometer tongue pressure manometer. Perspect ASHA SIGs.

[25] Potter NL, Short R (2009). Maximal tongue strength in typically developing children and adolescents. Dysphagia.

